# Application of TBSS-based machine learning models in the diagnosis of pediatric autism

**DOI:** 10.3389/fneur.2022.1078147

**Published:** 2023-01-18

**Authors:** Xiongpeng He, Xin Zhao, Yongbing Sun, Pengfei Geng, Xiaoan Zhang

**Affiliations:** ^1^Department of Imaging, Third Affiliated Hospital of Zhengzhou University, Zhengzhou, China; ^2^Henan International Joint Laboratory of Neuroimaging, Zhengzhou, China; ^3^Department of Imaging, People's Hospital of Zhengzhou University, Zhengzhou, China

**Keywords:** diffusion kurtosis imaging, autism, tract-based spatial statistics, back-propagation neural network, support vector machine, logistic regression

## Abstract

**Objective:**

To explore the microstructural changes of white matter in children with pediatric autism by using diffusion kurtosis imaging (DKI), and evaluate whether the combination of tract-based spatial statistics (TBSS) and back-propagation neural network (BPNN)/support vector machine (SVM)/logistic regression (LR) was feasible for the classification of pediatric autism.

**Methods:**

DKI data were retrospectively collected from 32 children with autism and 27 healthy controls (HCs). Kurtosis fractional anisotropy (FAK), mean kurtosis (MK), axial kurtosis (KA), radial kurtosis (RK), fractional anisotropy (FA), axial diffusivity (DA), mean diffusivity (MD) and Radial diffusivity (DR) were generated by iQuant workstation. TBSS was used to detect the regions of parameters values abnormalities and for the comparison between these two groups. In addition, we also introduced the lateralization indices (LI) to study brain lateralization in children with pediatric autism, using TBSS for additional analysis. The parameters values of the differentiated regions from TBSS were then calculated for each participant and used as the features in SVM/BPNN/LR. All models were trained and tested with leave-one-out cross validation (LOOCV).

**Results:**

Compared to the HCs group, the FAK, DA, and KA values of multi-fibers [such as the bilateral superior longitudinal fasciculus (SLF), corticospinal tract (CST) and anterior thalamic radiation (ATR)] were lower in pediatric autism group (*p* < 0.05, TFCE corrected). And we also found DA lateralization abnormality in Superior longitudinal fasciculus (SLF) (the LI in HCs group was higher than that in pediatric autism group). However, there were no significant differences in FA, MD, MK, DR, and KR values between HCs and pediatric autism group (*P* > 0.05, TFCE corrected). After performing LOOCV to train and test three model (SVM/BPNN/LR), we found the accuracy of BPNN (accuracy = 86.44%) was higher than that of LR (accuracy = 76.27%), but no different from SVM (RBF, accuracy = 81.36%; linear, accuracy = 84.75%).

**Conclusion:**

Our proposed method combining TBSS findings with machine learning (LR/SVM/BPNN), was applicable in the classification of pediatric autism with high accuracy. Furthermore, the FAK, DA, and KA values and Lateralization index (LI) value could be used as neuroimaging biomarkers to discriminate the children with pediatric autism or not.

## 1. Introduction

Autism spectrum disorder (ASD) is a specific type of pervasive developmental disorder characterized by deficits in social and verbal communication skills, and restricted repetitive behaviors (1). Some evidence demonstrates that the disrupted functional and structural connectivity will lead to these behavior features in ASD (2). Disrupted functional connectivity during language comprehension (3) and emotion recognition (4) had been reported in some ASD studies. Recently, more and more researches find there is association between abnormal functional connectivity and disrupted structural connectivity (5). Therefore, some cognitive dysfunction in ASD may be associated with disruption of white matter microstructure. In some cases, we can see deficiency in interhemispheric information transfer (6) and interhemispheric functional connectivity. To some extent, the impaired connectivity and abnormal lateralization of growth in the right and left brain may be related to the abnormal social and cognitive symptoms in ASD.

Nowadays, the early diagnosis of ASD is mainly based on the clinical symptoms, but most children with ASD have no typical clinical manifestations, which makes early diagnosis very difficult. However, some researches have reported appropriate systematic rehabilitation interventions in the early stages of autism could improve the symptoms of most patients (7, 8). It is widely accepted in the medical community that the earlier rehabilitation interventions are provided to children with ASD, the better prognosis will be. Therefore, it is particularly important for clinicians to make early and accurate diagnosis of pediatric autism.

Diffusion kurtosis imaging (DKI) is one of the state-of-the-art sequences that could be utilized to describe and sensitively detect microstructural changes of the complex brain tissues based on non-Gaussian water molecule theory (9). Tract-based spatial statistics (TBSS) is a statistical method to calculate the differences of diffusion images between patients and healthy children. The method enables objective spatial localization of group differences in DKI data, and its use of non-linear registration and full exploitation of the spatial determinants of major white matter bundles minimizes registration errors and biases and eliminates the need for arbitrary smoothing (10). Regarding disorders like ASD, TBSS can accurately locate the brain regions. A previous study combining DKI and TBSS to explore white matter abnormalities in the brain of adults with ASD confirmed that DKI could sensitively detect white matter abnormalities in the brain of patients with ASD and that a decrease in KA values reflected the severity of the patient, which was consistent with the findings of this study (11).

Machine learning methods, like support vector machine (SVM), have been applied to distinguish the children with mesial temporal lobe epilepsy with hippocampal sclerosis and healthy children (12). Recently, deep neural network, one new machine learning method has been attracting more and more attention in different fields, and it has been put into practice for classification of the brain disorders such as Alzheimer's disease (13, 14). Here, we try to use back-propagation neural network (BPNN) to do classification. BPNN has been widely used in different fields of scientific research (15) and has been demonstrated in several small sample studies (16, 17).

We hypothesize that there is one DKI parameter-based machine learning model can efficiently make a distinction whether a child has ASD or not. Firstly, we employed TBSS to explore the abnormalities of brain regions, and secondly, we combined the abnormal parameters of DKI and BPNN to make classification. Unlike the previous analysis using predefined ROI (18, 19), this study used TBSS for global analysis on DKI, and for the first time, the differences analyzed by TBSS were used as features for neural network training. To compare the performance of BPNN and traditional classifiers, we also employed the SVM (linear), SVM (RBF), logistic regression (LR) in python.

## 2. Materials and methods

### 2.1. Subject

All participants in our study were recruited in the Third Affiliated Hospital of Zhengzhou University between May 2020 and May 2021. Fifty-nine participants were retrospectively included into the study, including 32 children who were first diagnosed as ASD in the hospital and 27 healthy children. All patients were included based on the follow criteria: (1) 36–60 month old, right-handedness, (2) gestational age ≥37 weeks, (3) all patients met the ASD criteria of DSM-V (Diagnostic and Statistical Manual of Mental disorders-V), (4) the score of CARS (childhood autism rating scale) ≥30 points, (5) no history of head trauma and convulsion, no history of other mental diseases, no family history of neurological diseases, no history of psychotropic drug treatment, (6) no focal or diffuse lesions on imaging. All healthy controls (27 in total with matched age and gender) did not have any seizure and other developmental or neurological disorders.

### 2.2. MRI protocol

MRI acquisition was performed on 3T MR scanner (GE Signa Pioneer 3.0T). Thirty minutes before examination, all participants were given chloral hydrate (0.5 ml/kg) enemas. For stable image quality, earplugs with cotton balls were used to keep them from noise from the machine. After routine sequences scan, axial DKI was performed on all participants. The scanning parameters were: TR/TE = 8,200 ms/2.3 ms, FOV = 200 mm×200 mm, acquisition matrix = 256 × 256, NEX = 1, slices thickness = 4 mm, number of slices = 27; gradient values b: 0, 1,000 and 2,000 s/mm^2^; scanning time = 7 min 23 s. All subjects had no apparent structural damage, who were examined by two radiologists based on conventional MRI images.

### 2.3. Imaging preprocessing

All raw DKI image were processed on the iQuant workstation (GE Healthcare, Beijing, China). This workstation is an upgraded version of the commercial version of the software Horos (https://horosproject.org/). The latest AW4.7 version no longer supported the previous DKI processing platform, so GE's development team transferred the algorithms that included DKI image processing algorithm from the AW4.6 FuncTool platform to Horos and called it iQuant. After importing the images in the workstation, FA/MD/Da/DR and FAK/MK/KA/KR maps could be obtained automatically. We then exported the parameter images to a workstation on a VMware Linux virtual machine with FMRIB Software Library V5.0 (FSL, University of Oxford, UK) installed. FMRIB's Diffusion Toolbox (FDT), one tool of FSL, was employed to correct image distortions. Then, we used another tool of FSL named the Brain Extraction Tool (BET) to removed non-brain structure from the images.

### 2.4. TBSS analysis

Voxel-wise statistical analysis of FA/MD/DA/DR or FAK/MK/KR/KA maps were performed using TBSS (20), which was performed on FSL (21). Steps of TBSS analysis were as follows. First, each participant's FA image was registered with all other people's FA images, resulting in a study-specific template (i.e., tbss_2_reg-n). The found image that best represented the participants (i.e., the target image) was then affine-aligned into MNI152 standard space. All FA images were non-linearly transformed to the target image, and then the target image was affine transformed to MNI152 space, which finally made all FA images registered to MNI152 space. Then, the mean FA image of all participants was produced for the white matter skeleton with the FA threshold set to 0.2. Finally, this white matter skeleton was used as a binary mask. The FA/MD/DR/DA and FAK/MK/KR/KA images of each participant were projected onto the skeleton respectively, and subsequently exported to do voxel statistical analysis.

### 2.5. White matter lateralization indices

The DKI lateralization indices of each participant were generated from the original skeleton (asymmetric) and the symmetric skeleton that flipped the left side of the original skeleton to the right side. Their 4D prealigned data (all_FA or non-FA) was projected onto the symmetrized skeleton to generate the 4D file (all_FA_symmetrised_skeletonised or non-FA symmetrised_skeletonised). Next, we used the command (fslswapdim) to complete left-right switching of the 4D file to generate the flipped 4D file. The lateralization indices (LI) were calculated based on a formula as follows (22):

LI = (symmetrised_skeletonised– flipped) ÷ (symmetrised_ skeletonised + flipped).

### 2.6. Statistical analysis

We used the FSL randomize tool for statistical analysis, using 5,000 random permutations per test. Two contrasts were estimated: ASD greater than healthy controls and healthy controls greater than ASD. Considering that FA or other parameter maps might also be influenced by age or sex, we included age and sex as covariates for statistical analysis, trying to make the observed differences in parameters of DKI between groups independent. Threshold-free cluster enhancement (TFCE) (23) was used to calculate the significant differences between 2 groups with an initial threshold set at *p* < 0.05, after accounting for multiple comparisons by using family-wise error (FWE). Finally, we mapped the FWE-corrected statistical maps of *p* < 0.05 onto the JHU-WM Tractography Atlas in MNI space to localize and anatomically label the skeletal regions that showed significant differences in TBSS. Then we extracted the clusters detected in TBSS analysis and calculated their DKI parameter values as the raw features. Thereafter, principal component analysis (PCA) was used to select valid features from 59 participants, where 90% of the original information of the original features is retained.

The statistical analysis on baseline data was performed on IBM SPSS Statistics 25.0 (IBM Corporation, Armonk, NY, USA). We used independent t-test to compare the differences in continuous values (age). The Chi-square test was employed to compare the differences in counting values. The Kruskal-Wallis test was used for the comparison of Ordinal rank variables. *p* < 0.05 indicated statistical significance, and all statistical tests were two sided.

#### 2.6.1. Back-propagation neural network

**Figure 1:**
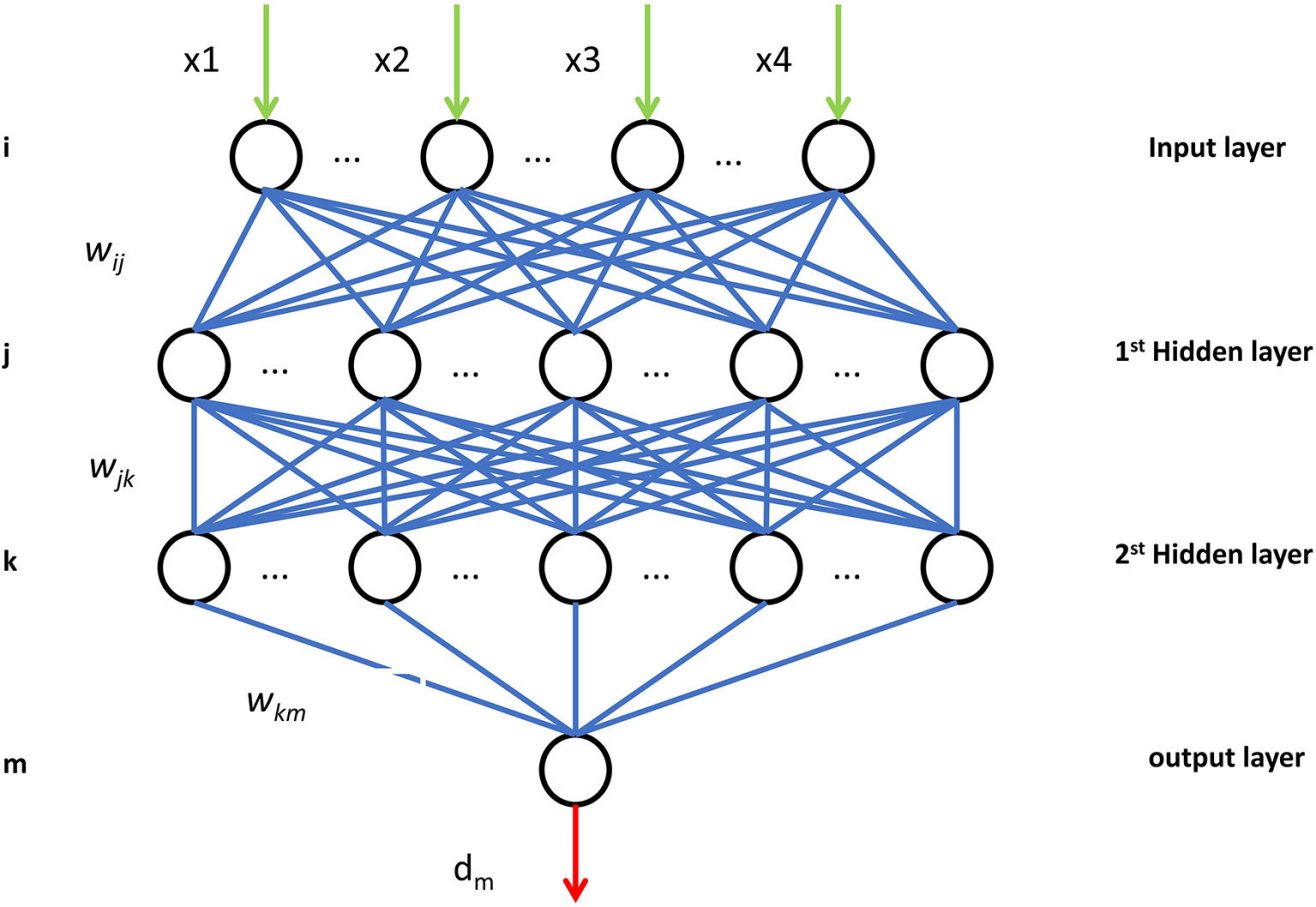
Structure diagram of four-layer BPNN. BPNN, back propagation neural network.

**Figure 2:**
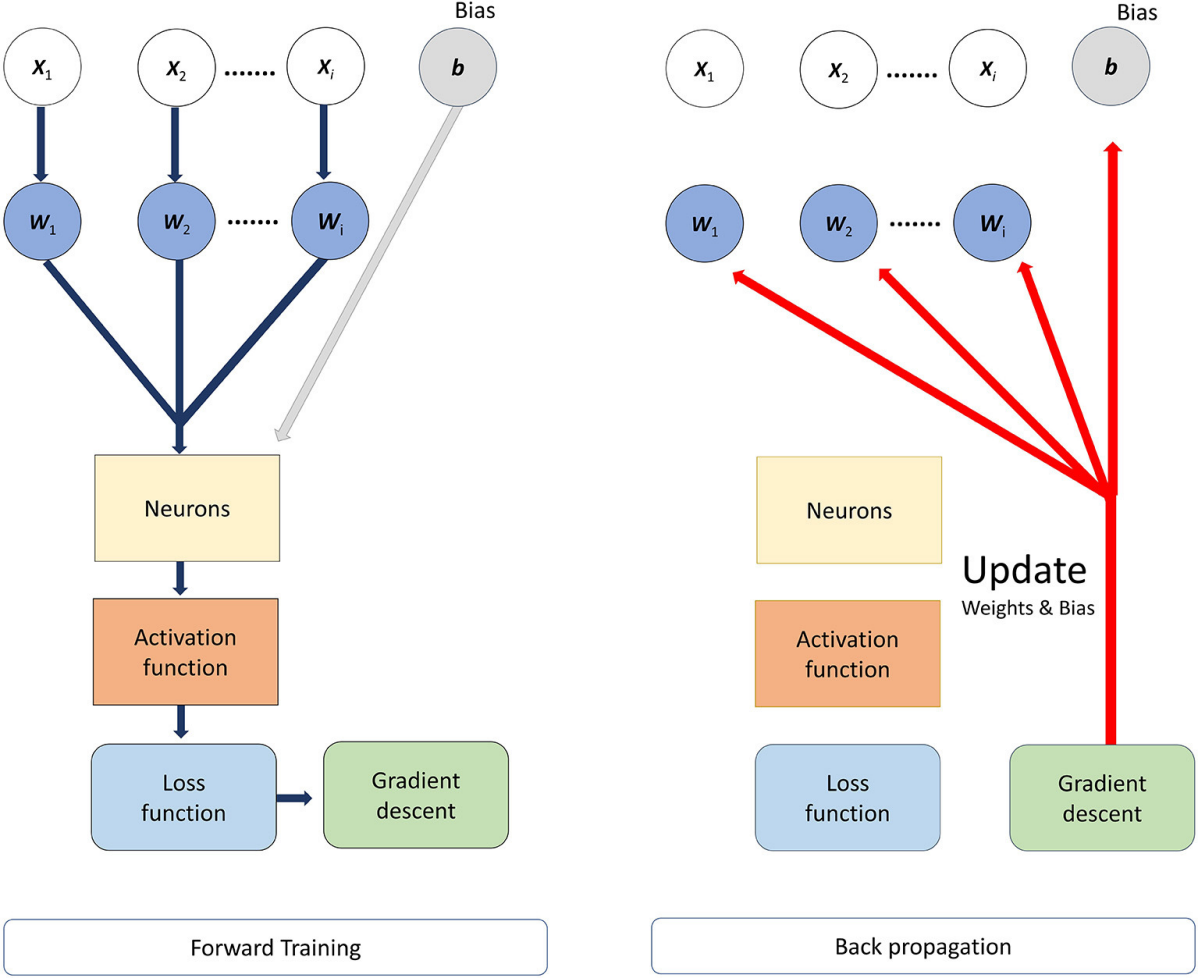
The workflow of backward propagation neural network (BPNN).

The Back-propagation network were conducted in python (version 3) using TensorFlow (version 1.2.1). In our case, the network was four-layer back-propagation (BP) neural network. The numbers of neurons in input layer and two hidden layers were 10, 16, and 8, while the numbers of neurons in output layer was 1. We adopted the hyperbolic tangent function (tanh) function as the activation function in hidden layer, and the Sigmoid function was adopted as activation function in output layer. For the loss function, binary cross entropy loss was adopted, which was


$$\begin{array}{l} Loss=-\frac{1}{N}\overset{N}{\underset{i=1}{\sum }}y_{i}\cdot log\left(p\left(y_{i}\right)\right)+\left(1-y_{i}\right)\cdot log\left(1-p\left(y_{i}\right)\right) \end{array}$$

where p(y) was the predicted value from network, whose range was 0 to 1. y was a label of the input subjects (y = 0 and y = 1) indicated the subjects belong to the Healthy control group (HC) and ASD group respectively, and the subscript *i* of y referred to the ordinal number of subjects (*i* = 1, 2, 3….., 59 in our research). Therefore, the loss value would be large if the subject belonged to ASD group (y = 1) and its prediction values [p(y)] was approaching 0, otherwise the loss value would be small. *N* was the total number of samples, and in our study, *N* = 59. Before the network was trained, the weights of the network are initialized from a Gaussian distribution with μ = 0, σ = 0.01 (μ was the mean value and σ was the standard deviation) and biases were initialized to 0. The batch size was 3, and then we trained the model for minimizing the values of loss function by Adam optimizer, of which the learning rate was setting at 0.001. To avoid the overfitting of the network, the L2 regularization was adopted, of which the parameter was setting at 0.01. L2 regularization enabled smaller values in the model and reduced model complexity; and we reduced the probability of overfitting by adding a dropout layer after each hidden layer; the dropout layer increased network diversity by randomly decreasing the number of neurons during training to avoid model overfitting, and in our study, the dropout layer parameter was set to 0.4 (40% of neurons were randomly deactivated in the hidden layers). The network structure is shown in Figure 1. The backward propagation neural network was trained by using multiple sets of data and continuously adjusting the weights of each link until the error of the network output reached the expected range. the process of BP neural network was divided into two main stages, the first stage was the forward propagation of the data, the input feature data passed from the input layer to the hidden layers and finally to the output layer, in this stage, the weights, bias values and activation functions were calculated, and the initial predicted values could be obtained. The second stage was the backward propagation of the error, from the output layer to the hidden layers and finally to the input layer; in this process, the error between the obtained initial prediction value and the target value was calculated, and then the gradient descent method was used to reduce the error and the weights and bias values would be updated. The workflow of BPNN is shown in Figure 2.

#### 2.6.2. Prediction and evaluation

To make full use of the subjects, leave-one-out cross validation (LOOCV) was adopted to estimate the classifier performance. In training steps, 58 subjects were used to training the network. And in testing steps, 1 subject would be used to evaluate the trained model. Finally, a total of 59 repetitions of the above loop were required to predict the participants' labels. The accuracy, specificity, sensitivity and F1-score of models were used to quantify the performance of the model, which were calculated as:


$$\begin{array}{lll} Accuracy & = & \frac{TP+TN}{TP+FP+TN+FN}\\ Sensitivity & = & \frac{TP}{TP+FN}\\ Specificity & = & \frac{TN}{TN+FP}\\ Precision & = & \frac{TP}{TP+FP}\\ Recall & = & \frac{TP}{TP+FN} \end{array}$$

$$\begin{array}{lll} F1-Score & = & 2\;*\;\frac{Precision\;*\;Recall}{Precision+Recall}\; \end{array}$$

where TP, TN, FP, and FN meant true positive, true negative, false positive, and false negative respectively.

#### 2.6.3. Comparison with traditional classifiers

For comparing the BPNN against traditional classifiers, the support vector machine (SVM) with radial basis function (RBF) and linear kernel and Logistic Regression (LR) model were performed in python.

##### 2.6.3.1. Support vector machine

Support vector machines are model-based learning algorithms that perform classification mainly by employing a hyperplane. The hyperplane can be constructed using kernel functions such as linear, radial basis functions (RBF), etc. In our research, we mainly used linear as well as RBF. In SVM, there is an important parameter C (the penalty coefficient); the higher the C, the more unacceptable appears the error, meaning easy to overfit; and the smaller the C, easy to underfit. For the RBF, there is another important parameter g (gamma), which determines the distribution of the data after mapping to the new feature space.

##### 2.6.3.2. Logistic regression

Logistic regression is a widely used model for data classification, and it is often used in binary classification. It builds a probabilistic model that calculates the probability that the output variable y will be 0 or 1 given the input variable x. In this model, there is an important parameter C′ (regularization coefficient). It is inversely proportional to the penalty coefficient, which means that the smaller the value of C′, the stronger the regularization effect and the greater the penalty effect on the parameters. And in our experiments, we also experimented with the four solvers (liblinear, lbfgs, newton-cg, and sag) to find the best combination of parameters using grid search.

In order to optimize the classifier parameters, we performed a grid search for all the hyperparameters of the all models (BPNN/LR/SVM). For SVM and LR, there are two parameters to optimize (SVM: C and g; LR: C′ and solvers). And for BPNN, there are six parameters to be optimized (the numbers of neurons, epochs, batch size, learning rate, L2 value, and the values of drop-out layers). The hyperparameters and their values are shown in Supplementary Table 8. The LOOCV is used to select the combination of hyperparameters with the highest model accuracy.

**Figure 3:**
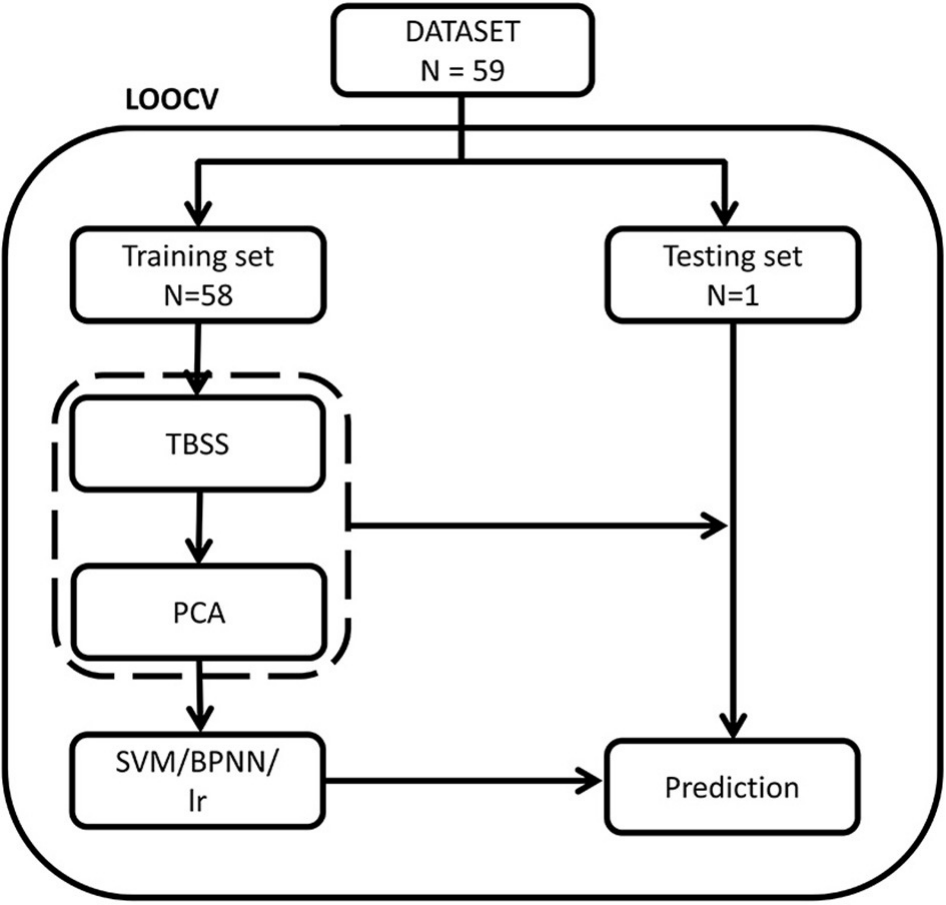
The flowchart of leave-one-out cross validation. PCA, principal component analysis; SVM, support vector machine; LR, logistic regression; TBSS, tract-based spatial statistics.

The training steps of the traditional models were identical to the BPNN. Then, the LOOCV in traditional models would repeat 59 times (*n* = 59) to evaluate the performance of the models. We also calculated the receiver operating characteristic (ROC) curve for all models and compared the area under the ROC curve (AUC) of all models by using Delong's test (24) to evaluate the performance of different models. The McNemar's test was used to compare the differences of their accuracy, sensitivity and specificity. The platforms of the SVM (linear or RBF) and LR were python using Scikit-learn library (version 0.24.2), while BP neural network was performed using TensorFlow (1.2.1). The flowchart of leave-one-out cross validation (LOOCV) was shown in Figure 3.

## 3. Result

### 3.1. Demographic characteristics

At baseline, there were no statistical difference in gender, age, their birth way, their parents' educational background, and their feeding pattern (*p* > 0.05, Table 1).

**Table 1:** Two groups demographic characteristics.

**Characteristics**	**ASD patients**	**HC**	***p***
Age (mean ± STD) (weeks)	34.12 ± 9.15	29.96 ± 11.52	0.128^a^
Gender (males/females)	23M/9F	19M/8F	0.899^b^
Birth way (eutocia/abdominal delivery)	38.44 ± 1.56	37.56 ± 2.04	0.060^b^
Educational background (mom)	③ = 0%	③ = 7%	0.938^c^
	④ = 53.1%	④ = 41%	
	⑤ = 46.9%	⑤ = 52%	
Educational background (father)	③ = 0%	③ = 3.5%	0.440^c^
	④ = 37.5%	④ = 44.5%	
	⑤ = 62.5%	⑤ = 52%	
Feeding pattern	A = 53%	A = 29.6%	0.358^c^
	B = 12.5%	B = 25.4%	
	C = 34.5%	C = 45%	

a Independent sample t-test.

b Chi-square test.

c Kruskal-Wallis test.

Feeding pattern: A = breast feeding, B = formula feeding, C = mixed feeding (breast milk and formula).

Educational background (mother/father): ③ = El ementary school degree and below; ④ = Middle School–High School Degree; ⑤ = Bachelor degree and above.

### 3.2. TBSS analysis results

**Figure 4:**
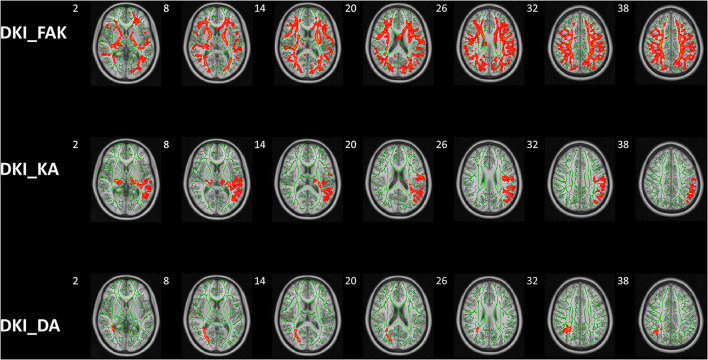
Group differences of TBSS in FAK, KA, and DA. Tract-based spatial statistics shows white matter regions with significant (*p* < 0.05, threshold-free cluster enhancement corrected) differences in FAK, KA, and DA values between ASD children and HCs. Green represents mean FA skeleton of all participants; red denotes parameters reduction in ASD children. DKI, diffusion kurtosis imaging; HCs, healthy controls; KFA, kurtosis FA; DA, axial diffusivity; KA, axial kurtosis.

The significant clusters of subjects in Kurtosis fractional anisotropy are summarized in Table 2. Reduced Kurtosis fractional anisotropy (FAK) was observed in the bilateral CST, ATR, CG, HIP, IFOF, ILF, SLF, and UF, the temporal part of SLF as well as the FMA and FMI in ASD group in contrast to the healthy control group. We also found the ASD group showed significant axial kurtosis (KA) decrease in the bilateral CST, ATR, IFOF and the left SLF, ILF, UF as well as the left temporal part of SLF relative to the healthy control group (Supplementary Table 1). Compared to the healthy controls, the ASD group showed significant axial diffusivity (DA) decrease in FMA and the right ILF, IFOF, SLF, ATR as well as the right temporal part of SLF (Supplementary Table 2). In addition, the lateralization index (LI) of ASD group in the left SLF showed significant increase compared with healthy control group (Supplementary Table 3). No significant between group differences were observed in fractional anisotropy (Fa), radial diffusivity (RD), mean diffusivity (MD) and mean kurtosis (MK), radial kurtosis (KR). Figure 4 illustrated the group differences for the three parameters.

**Table 2:** Between-group differences in Kurtosis fractional anisotropy.

**Comparison** **(FAK)**	**Cluster no**.	**Anatomical regions**	**Number of** **voxels**
HC > ASD	1	Left ATR	18
		Left IFOF	
	2	Right CG	22
	3	Left HIP	23
	4	Left SLF	27
	5	Left ATR	160
		Left IFOF	
		Left SLF	
		Left UF	
	6	Right ILF	472
		Right SLF	
		Right SLF (temporal part)	
	7	Left ATR	4,503
		Right ATR	
		Left CST	
		Right CST	
		Left Cingulum (CG)	
		Right Cingulum (CG)	
		Left Cingulum (HIP)	
		Right Cingulum (HIP)	
		FMA	
		FMI	
		Left IFOF	
		Right IFOF	
		Left ILF	
		Right ILF	
		Left SLF	
		Right SLF	
		Left UF	
		Right UF	
		Left SLF (temporal part)	
		Right SLF (temporal part)	

### 3.3. Prediction performance

**Figure 5:**
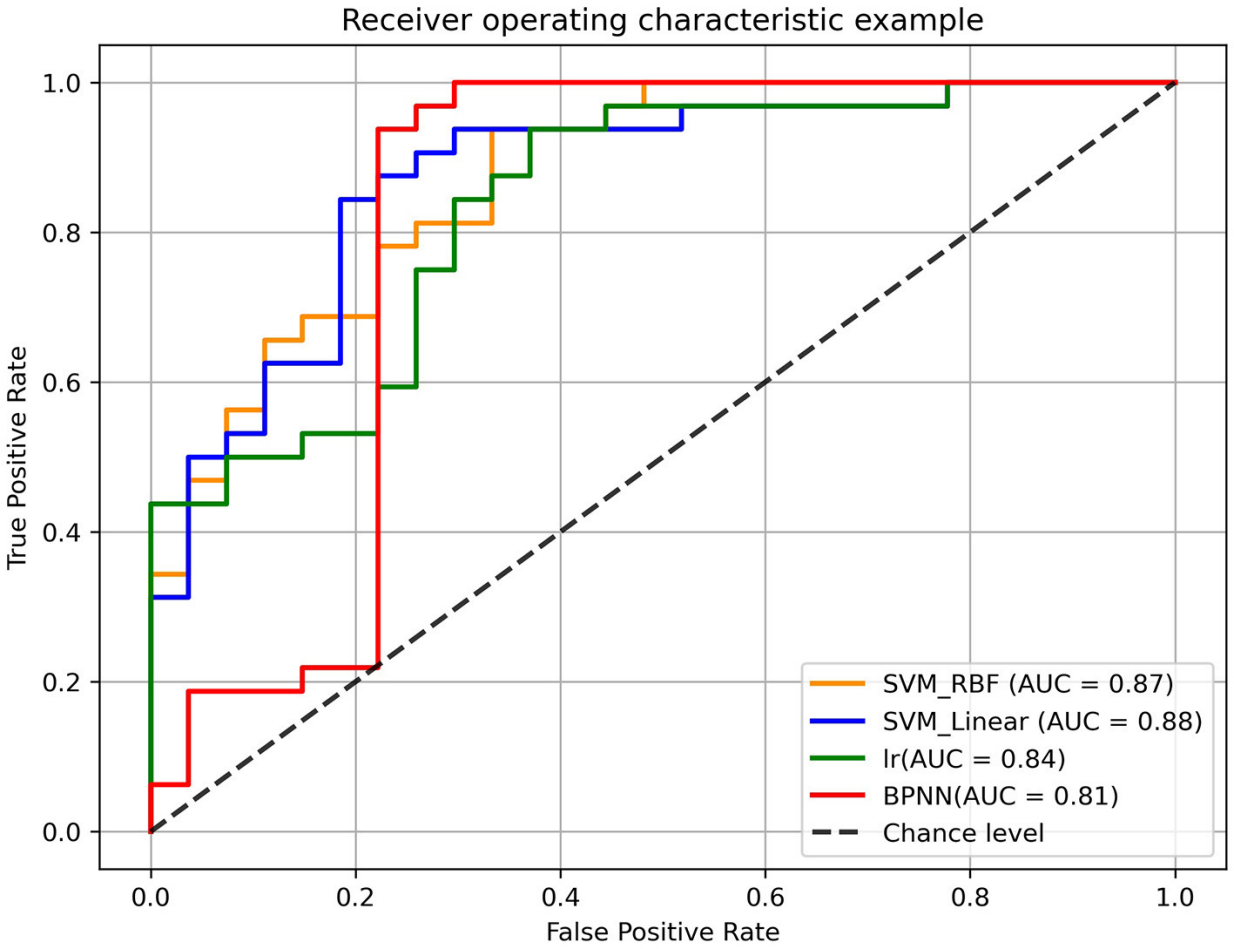
The receiver operating characteristic (ROC) curves of different models. The orange curves, blue curves, green curves, and red curves represented the roc curve of SVM (RBF Kernel), SVM (linear Kernel), logistic regression (LR) model and back-propagation neural network (BPNN) model, respectively.

The performance of all models were shown in Table 3. In LOOCV experiment, the accuracy, sensitivity and specificity of BPNN were 86.44%, 96.88%, and 74.07% respectively. The AUC reached 0.81, indicating that BPNN had good classification performance (Table 3). Then, there was no difference between the different models in the comparison of specificity and sensitivity, but we can find that there is a significant difference between BPNN and LR in accuracy (McNemar's test, *p* < 0.05) (Supplementary Table 5). In addition, there was no significant difference between BPNN and SVM in accuracy (McNemar's test, *p* > 0.05). We also performed Delong' test to do comparison on different models' AUC values, which shown no statistical differences in AUC between different models (Delong's test, *p* > 0.05) (Supplementary Table 4). The ROC curves were shown in Figure 5. And we could find that in the F1 score, BPNN had the best result, reaching 88.89%, while the F1 score of LR was lowest, which was only 79.41%.

**Table 3:** The evaluation indicators of SVM (linear), SVM (RBF), BPNN and LR.

**Indicators**	**Models**			
	**SVM (RBF)**	**SVM (linear)**	**LR**	**BPNN**
Accuracy	81.36%	84.75%	76.27%	86.44%
Sensitivity	93.75%	90.36%	84.38%	96.88%
Specificity	66.67%	77.78%	66.67%	74.07%
AUC	0.86	0.88	0.84	0.81
F1-score	84.51%	86.57%	79.41%	88.89%

SVM, support vector machine; RBF, radial basis function; BPNN, back-propagation neural network; LR, logistic regression; AUC, area under the curve.

## 4. Discussion

In clinical practice, most routine MRI findings are negative, which is detrimental to the early diagnosis of autism (25, 26). However, DKI, a kind of diffusion-weighted imaging, can provide abundant physiologic information about brain tissues (27). Different with conventional diffusion-weighted imaging, DKI imaging is a recently developed method that has power to measure the non-Gaussian diffusion, which makes DKI suitable to investigate the micro-structure changes (28).

### 4.1. Major altered parameters and their clinical significance

In our study, TBSS, an automated analysis based on tract, was adopted to study micro-structure changes of white matter fiber tracts in children with ASD. Decreased FAK, KA, and DA in extensive fiber bundles were observed in ASD group compared to healthy control group, indicating widespread white matter damage in children with ASD. We also found that the children with ASD show increased LI in DA in SLF compared to healthy control group, indicating developmental abnormalities of brain lateralization in children with ASD.

FAK is like FA to some extent measuring anisotropy but adopts the kurtosis tensor for calculation, which leads to less errors from complex white matter fiber arrangements (29). When the bulk density of fiber bundles and axons increases, it can also provide the necessary supplement for poor FA performance (30). Notwithstanding, lots of studies did not adopt the FAK parameter to evaluate the micro-structure changes. However, we found decreased FAK in extensive fiber tracts of children with ASD in our study. Previous studies also proved FAK might be a significant parameter evaluating white matter micro-structure changes (31, 32). Compared to FAK, FA parameter was widely used in assessing white matter with coherent fiber arrangements, such as CST. But for the crossing fibers, such as ILF, FA has limited evaluation value. In our results, multiple complex fiber alignment changes were found, which may explain why FA is not sensitive in detecting white matter damage, but FAK is. The decrease in FAK in multiple fiber tracts shows clear signs of white matter axonal damage, which suggests those regions are indeed involved in ASD.

The KA and DA parameters of DKI are also commonly used kurtosis and diffusion metrics. KA is a directional kurtosis parameter parallel to the long axis of the diffusion tensor, which reflects the integrity of the axon. DA is a metric of diffusion along the axis, which was proved to be a biomarker of axon damage (33). In our findings, we found a decreased in DA in the right side of IFOF, ILF and SLF, while decrease in KA was observed in more regions. In addition, the cluster sizes of altered KA were larger than those of DA values. Some regions such as CST and UF showed KA alteration but without DA alterations. To some extent, it shows that the kurtosis parameter is more sensitive than the diffusion parameter. The destruction of myelin integrity, nerve fiber density and parallelism and disorder of a microstructure may contribute directly to the decreased KA and DA. And our results are consistent with previous studies in terms of the trend of KA changes (31).

Previous studies have shown that children with ASD have thalamic-frontal connectivity disorders (34). In addition, previous studies have reported a reduction in thalamic volume in patients with ASD (35) and a loss of linear relationship between thalamic volume and whole brain volume (36), which also indirectly confirmed the presence of cortico-thalamic connectivity damage in children with ASD. Decreased FAK, DA, and KA were observed in bilateral ATR, which is consistent with previous studies. Besides, we also found the changes of DKI parameters in bilateral ILF and IFOF, with the IFOF being associated with language processing (37) and the ILF being associated with reading ability (38). This also explains the symptoms of reading as well as verbal communication difficulties in children with ASD in our case. UF is a bundle of medial white matter fibers that connects the temporal lobe (including the amygdala and HIP) to the insula and orbitofrontal cortex. And it mediates ventral limbic connections and facilitates integration between structures that process emotional and cognitive information (39). The impairment of UF may be associated with deficits in social-emotional processing in ASD, resulting in a lack of empathy in children with ASD.

Currently, our study is the first to use DKI to study brain lateralization in children with ASD. Because of its ability to assess human growth and development, the lateralization index is widely used in different fields, such as behavioral assessment (40). In our results, the DA lateralization index was significantly higher in children with ASD than in children with normal development; furthermore, we found that the reduction of DA parameters in children with ASD occurred only in the right side of the brain during the differential comparison, while the DA parameters in the left side of the brain were not dissimilar to those of normal children. This suggests that the increase in DA lateralization index in children with ASD might be due to the decrease in DA values in the right side of the brain. As we know, the central nervous system (from the brain) crosses into two bundles at the cervical vertebrae (neck) to govern the left and right limbs, so there is a directional crossover between the cerebral hemispheres and limb behavior, for example, the right brain mainly governs the left limb movement. Also, according to previous reports, there is an increased rate of left-handedness in children with ASD (41). Catani et al. (42) used the DTI technique to demonstrate that the SLF is the link between anterolateral, posterior and lateral language areas of the white matter pathway. In terms of language function, the superior longitudinal tract transfers the brain's understanding to the dominant hemisphere for expression (also known as “language transfer”) (43, 44). Some studies have reported that lesions of the superior longitudinal tract could also cause conduction aphasia (45). However, most of the children with ASD in our case would have problems with language impairment, our results explained this symptom to some extent. Overall, the DA lateralization index may be a suggestive indicator of altered language skills as well as motor skills in children with early ASD.

### 4.2. Performance of different models

Our results of TBSS on DKI image might provide efficient diffusion and kurtosis features as a possible biomarker for classification models based on machine learning methods. Most previous brain science research on classification has focused on the predefined ROI changes (18, 19). However, the performance of predefined features within the classifier is often too subjective, resulting in unreliable performance. In our study, our features were based on a whole-brain differential comparison and the differential parameters were consistent with the patients' clinical symptoms. Thus, the features we provide to the classifier are objective, and the features have better performance in sensitivity and specificity.

We tried to use BPNN for classification, which was better than LR in terms of accuracy, although not much different from SVM accuracy. We also compared the specificity and sensitivity as well as the AUC values of the four models, and although the differences were not statistically significant (Supplementary Tables 6, 7), the sensitivity of BPNN was quite high, with outstanding performance in the detection of cases. We found that although BPNN did not perform well in terms of AUC values, BPNN was basically higher than the rest of the traditional classifiers in terms of F1 scores; we suspected that this is due to the unbalanced data (There were more subjects in the ASD group than in the HC group.); previous study had shown that F1 scores better reflected the actual performance of classifiers under the premise of unbalanced data (46), and this further confirms that BPNN combined with the TBSS classifier could produce better classification performance. Due to our relatively small sample size, we used SVM, LR and BPNN models that were relatively robust to the overfitting phenomenon (47), and one previous study showed that LR and SVM were suitable for small samples size research. Secondly, we introduced LOOCV, which avoided overfitting to some extent by bias-variance trade-off. Therefore, in combination with LOOCV and the corresponding model, the reliability of our results was confirmed.

### 4.3. Limitation

There are some potential limitations in our study. First, this study had a small sample size and lacked validation with independent datasets. More large multi-center datasets need to be assessed in the future to confirm the results. Second, the imaging data is single, and the mechanism behind it can be more effectively elucidated if multiple imaging modalities are combined, including fMRI such as resting-state functional MRI. However, for children, the acquisition of imaging data is still a challenge. Although our method achieves an acceptable level of accuracy, it is still not a substitute for traditional diagnostic methods, and the future combination of clinical indicators as well as the combination of multidisciplinary indicators will facilitate further confirmation of our study.

## 5. Conclusion

Our study shows that the combination of TBSS of DKI and machine learning methods might be effective in discriminating the children with ASD or not. Furthermore, FAK, KA, and DA parameters and LI values have the potential to be used as biomarkers to differentiate children with early ASD from those with normal development. Future studies with large and multi-center samples may be helpful to further elucidate the DKI parameters and the underlying neurobiological mechanisms, and our proposed method may be useful for future multidisciplinary diagnosis of early ASD.

## Data availability statement

The raw data supporting the conclusions of this article will be made available by the authors, without undue reservation.

## Ethics statement

The studies involving human participants were reviewed and approved by the Ethics Committee of the Third Affiliated Hospital of Zhengzhou University. Written informed consent to participate in this study was provided by the participants' legal guardian/next of kin.

## Author contributions

XH: experimental design, paper writing, and statistics. YS and XH: data acquisition. XZhang and XZhao: experiment design and funding provision. PG: language modifications. All authors contributed to the article and approved the submitted version.
