# Supplementary material for: Application of TBSS-based machine learning models in the diagnosis of pediatric autism
Source: Front Neurol. 2023 Jan 18;13:1078147. doi: 10.3389/fneur.2022.1078147 (PMC9889873; doi:10.3389/fneur.2022.1078147)
Supplement: Supplementary file 1 [file Table_1.DOCX]

***Supplementary materials***

**S-Table 1 Between-group differences in axial kurtosis (KA)**

| Comparison  （KA） | Cluster No. | Anatomical regions | Number of voxels |
| --- | --- | --- | --- |
| HC > ASD | 1 | Right ATR | 10 |
|  | 2 | Right ATR  Right CST | 17 |
|  | 3 | Left SLF  Left SLF (temporal part) | 22 |
|  | 4 | Left ATR  Left CST | 25 |
|  | 5 | Left ATR  Left CST  Right CST | 29 |
|  | 6 | Left ATR | 30 |
|  | 7 | Left SLF | 31 |
|  | 8 | Right CST | 32 |
|  | 9 | Right ATR  Right CST | 73 |
|  | 10 | Right ATR  Right CST | 77 |
|  | 11 | Left ATR  Left CST | 162 |
|  | 12 | Left SLF | 178 |
|  | 13 | Left SLF | 199 |
|  | 14 | Right ATR  Right CST  Right IFOF | 236 |
|  | 15 | Left SLF  Left SLF (temporal part) | 352 |
|  | 16 | Left ATR  Left CST  Left IFOF  Left ILF  Left SLF  Left UF  Left SLF (temporal part) | 6742 |

**S-Table 2 Between-group differences in axial diffusivity (DA)**

| Comparison  （DA） | Cluster No. | Anatomical regions | Number of voxels |
| --- | --- | --- | --- |
| HC > ASD | 1 | FMA  Right IFOF  Right ILF  Right SLF | 319 |
|  | 2 | Right ATR  FMA  Right IFOF  Right ILF  Right SLF  Right SLF (temporal part) | 534 |

**S-Table 3 Between groups differences in lateralization index (LI)**

| Comparison  （IL）(DA) | Cluster No. | Anatomical regions | Number of voxels |
| --- | --- | --- | --- |
| HC< ASD | 1 | SLF | 30 |

**S-Table 4 The results of Delong's Test between different models**

| Comparisons of AUC | Z values | P values |
| --- | --- | --- |
| BPNN vs LR | -0.809 | 0.418 |
| SVM(L) vs SVM(R) | -0.909 | 0.363 |
| SVM(R) vs LR | 1.212 | 0.225 |
| SVM(R) vs BPNN | 1.226 | 0.220 |
| SVM(L) vs LR | 1.439 | 0.150 |
| SVM(L) vs BPNN | 1.609 | 0.107 |

**S-Table 5 The results in accuracy of McNemar's Test between different models**

| Comparisons of accuracy | Statistic values | P values |
| --- | --- | --- |
| SVM(L) vs SVM(R) | 2 | 0.688 |
| SVM(R) vs LR | 1 | 0.375 |
| SVM(R) vs BPNN | 0 | 0.250 |
| SVM(L) vs LR | 1 | 0.125 |
| SVM(L) vs BPNN | 2 | 1 |
| BPNN vs LR | 0 | 0.031***** |

Note: * indicates a statistical difference between the two models in accuracy.

**S-Table 6 The results in sensitivity of McNemar's Test between different models**

| Comparisons of sensitivity | Statistic values | P values |
| --- | --- | --- |
| SVM(L) vs SVM(R) | 1 | 1 |
| SVM(R) vs LR | 1 | 0.375 |
| SVM(R) vs BPNN | 0 | 0.5 |
| SVM(L) vs LR | 1 | 0.625 |
| SVM(L) vs BPNN | 0 | 0.25 |
| BPNN vs LR | 0 | 0.063 |

**S-Table 7 The results in specificity of McNemar's Test between different models**

| Comparisons of specificity | Statistic values | P values |
| --- | --- | --- |
| SVM(L) vs SVM(R) | 0 | 0.25 |
| SVM(R) vs LR | 0 | 1 |
| SVM(R) vs BPNN | 0 | 1 |
| SVM(L) vs LR | 0 | 0.25 |
| SVM(L) vs BPNN | 0 | 0.50 |
| BPNN vs LR | 0 | 1 |

**S-Table 8 Hyperparameters of the classifiers and the range of their values**

| Classifiers | Hyperparameters | Values |
| --- | --- | --- |
| SVM(L) | C | [1,10] |
| SVM(R) | C | [1,10] |
|  | g | [0.1,1] |
| LR | C’ | [0.1,20] |
|  | solvers | liblinea，lbfgs，newton-cg，sag |
| BPNN | Hidden layer1  Hidden layer2  Batch size  Epochs  Drop-out layers  L2  Learning rate | 16,8,4  16,8,4  3,6,9,12,15,18,21  2000,3000,4000,5000  0.2,0.4,0.6,0.8  0.1,0.01,0.001,0.0001  0.1,0.01,0.001,0.0001,0.00001 |
